# The European Society of Gynaecological Oncology (ESGO), the International Society for the Study of Vulvovaginal Disease (ISSVD), the European College for the Study of Vulval Disease (ECSVD), and the European Federation for Colposcopy (EFC) Consensus Statement on the Management of Vaginal Intraepithelial Neoplasia

**DOI:** 10.1097/LGT.0000000000000732

**Published:** 2023-03-23

**Authors:** Vesna Kesic, Xavier Carcopino, Mario Preti, Pedro Vieira-Baptista, Federica Bevilacqua, Jacob Bornstein, Cyrus Chargari, Maggie Cruickshank, Emre Erzeneoglu, Niccolò Gallio, Murat Gultekin, Debra Heller, Elmar Joura, Maria Kyrgiou, Tatjana Madić, François Planchamp, Sigrid Regauer, Olaf Reich, Bilal Esat Temiz, Linn Woelber, Jana Zodzika, Colleen Stockdale

**Affiliations:** 1 Medical Faculty, University of Belgrade, Clinic of Obstetrics and Gynecology, University Clinical Center of Serbia, Belgrade, Serbia; 2 Department of Obstetrics and Gynaecology, Hôpital Nord, APHM, Aix-Marseille University (AMU), Univ Avignon, CNRS, IRD, IMBE UMR 7263, 13397, Marseille, France; 3 Department of Surgical Sciences, University of Torino, Torino, Italy; 4 Lower Genital Tract Unit Centro Hospitalar de São João, Porto, Portugal; 5 Hospital Lusiadas, Porto, Portugal; 6 Galilee Medical Center and Azrieli Faculty of Medicine, Bar-Ilan, Israel; 7 Department of Radiation Oncology, Gustave Roussy Cancer Campus, Villejuif, France; 8 Aberdeen Centre for Women’s Health Research, University of Aberdeen, Aberdeen, United Kingdom; 9 Faculty of Medicine, Department of Obstetrics and Gynecology, Division of Gynaecological Oncology, Hacettepe University, Ankara, Turkey; 10 Division of Gynaecological Oncology, Department of Obstetrics and Gynaecology, Hacettepe University Faculty of Medicine, Ankara, Turkey; 11 Rutgers New Jersey Medical School, Newark, NJ; 12 Department of Gynecology and Gynecologic Oncology, Comprehensive Cancer Center, Medical University of Vienna, Vienna, Austria; 13 Surgery and Cancer - West London Gynecological Cancer Center, IRDB, Department of Gut, Metabolism & Reproduction-Surgery & Cancer, Imperial College London, London, United Kingdom; 14 Imperial Healthcare NHS Trust, Queen Charlotte's & Chelsea Hospital West London Gynaecological Cancer Centre, London, United Kingdom; 15 Clinic for Obstetrics and Gynecology, University Clinical Center of Serbia, Belgrade, Serbia; 16 Clinical Research Unit, Institut Bergonie, Bordeaux, France; 17 Diagnostic and Research Institute of Pathology, Medical University of Graz, Graz, Austria; 18 Department of Obstetrics and Gynecology, Medical University of Graz, Graz, Austria; 19 Department of Gynecology, Hamburg-Eppendorf University Medical Center, Hamburg, Germany; 20 Dysplasia Center Hamburg; Jerusalem Hospital Hamburg, Hamburg, Germany; 21 Department of Obstetrics and Gynaecology Rīga Stradiņ,š University, Riga, Latvia; 22 Department of Obstetrics & Gynecology, University of Iowa, Iowa City, IA

## Abstract

The European Society of Gynaecological Oncology (ESGO), the International Society for the Study of Vulvovaginal Disease (ISSVD), the European College for the Study of Vulval Disease (ECSVD), and the European Federation for Colposcopy (EFC) developed consensus statements on pre-invasive vulvar lesions in order to improve the quality of care for patients with vaginal intraepithelial neoplasia (VaIN). The management of VaIN varies according to the grade of the lesion: VaIN 1 (low grade vaginal squamous intraepithelial lesions (SIL)) can be subjected to follow-up, while VaIN 2–3 (high-grade vaginal SIL) should be treated. Treatment needs individualization according to the patient’s characteristics, disease extension and previous therapeutic procedures. Surgical excision is the mainstay of treatment and should be performed if invasion cannot be excluded. Total vaginectomy is used only in highly selected cases of extensive and persistent disease. Carbon dioxide (CO_2_) laser may be used as both an ablation method and an excisional one. Reported cure rates after laser excision and laser ablation are similar. Topical agents are useful for persistent, multifocal lesions or for patients who cannot undergo surgical treatment. Imiquimod was associated with the lowest recurrence rate, highest human papillomavirus (HPV) clearance, and can be considered the best topical approach. Trichloroacetic acid and 5-fluorouracil are historical options and should be discouraged. For VaIN after hysterectomy for cervical intraepithelial neoplasia (CIN) 3, laser vaporization and topical agents are not the best options, since they cannot reach epithelium buried in the vaginal scar. In these cases surgical options are preferable. Brachytherapy has a high overall success rate but due to late side effects should be reserved for poor surgical candidates, having multifocal disease, and with failed prior treatments. VaIN tends to recur and ensuring patient adherence to close follow-up visits is of the utmost importance. The first evaluation should be performed at 6 months with cytology and an HPV test during 2 years and annually thereafter. The implementation of vaccination against HPV infection is expected to contribute to the prevention of VaIN and thus cancer of the vagina. The effects of treatment can have an impact on quality of life and result in psychological and psychosexual issues which should be addressed. Patients with VaIN need clear and up-to-date information on a range of treatment options including risks and benefits, as well as the need for follow-up and the risk of recurrence.

## BACKGROUND

Vaginal intraepithelial neoplasia (VaIN) is difficult to diagnose and manage and has substantial potential to evolve to invasive cancer. It is a rare disease, but as some patients are at increased risk, knowledge of the epidemiology, natural history, diagnosis, and treatment of VaIN is highly important for prevention of invasive vaginal cancer.

The European Society of Gynaecological Oncology (ESGO), the International Society for the Study of Vulvovaginal Disease (ISSVD), the European Federation for Colposcopy (EFC), and the European College for the Study of Vulval Disease (ECSVD) are leading international societies among gynecologists, pathologists, dermatologists, and other related disciplines. ESGO, ISSVD, EFC, and ECSVD have agreed to collaborate in order to produce a consensus statement on the management of pre-invasive vulvar and vaginal lesions. A consensus statement on the management of pre-invasive vulvar disease has been published already.^1^ This consensus statement focuses on the management of VaIN. The statement was accepted when consensus of at least two thirds of experts was achieved.

## METHODS

The ESGO, ISSVD, EFC, and ECSVD Executive Councils nominated specialists among their members, whose expertise in improving the quality of care for patients with vaginal pre-invasive lesions has been previously confirmed. Five residents were invited to summarize the evidence available. Two external experts, internationally acknowledged for their research in vaginal pre-invasive lesions, were invited to review the final manuscript, before submission and external peer review.

A systematic literature review of the studies published from January 2000 to April 2022 was carried out using the MEDLINE database. Search indexing terms and criteria are listed in an additional file (see Supplemental Digital Content 1, https://dx.doi.org/10.1136/ijgc-2022-004213). Priority was given to high-quality systematic reviews, meta-analyses, and randomized controlled trials. The search strategy excluded editorials, case reports, letters, and *in vitro* studies.

A total of 97 articles were retrieved dealing with VaIN. Data extraction was performed for all the articles on treatment by two independent teams with double-checking to ensure completeness. Tables with the most relevant clinical outcomes of 54 studies related to the treatment of VaIN were completed and summarized in the text (see Supplemental Digital Content 2, https://dx.doi.org/10.1136/ijgc-2022-004213). The other sections of this paper were drafted by one or more authors, with an independent literature search. A consensus was achieved between all the authors concerning the final version of the document.

Evidence-based consensus statements were also developed on the management of patients with VaIN, chaired by Vesna Kesic. The chair was responsible for drafting corresponding preliminary statements based on the review of the relevant literature (residents assisted in preparing data extraction and analyses: F.B., N.G., B.E.E., B.E.T.). These were then sent to the group of selected specialists. A first round of binary voting (agree/disagree) was carried out for each potential statement. The participants took part in each vote, but they were permitted to abstain from voting if they felt they had insufficient expertise to agree/disagree with the statement or if they had a conflict of interest that could be considered to influence their vote. The voters had the opportunity to provide comments/suggestions with their votes. The chairs then discussed the results of this first round of voting and revised the statements if necessary. The voting results and the revised version of the statements were again sent to the whole group and another round of binary voting was organized, according to the same rules, to allow the whole group to evaluate the revised version of the statements. The statements were finalized based on the results of this second round of voting. The group achieved consensus on 13 statements. One of the authors (F.P.) provided the methodology support for the entire process and did not participate in voting for statements.

Two external independent reviewers, internationally acknowledged for their research in VaIN, reviewed the final manuscript (M.K., S.R.).

Given the characteristics of this study, no ethical approval was considered necessary.

### Evolution of Terminology and Classifications

In 2012, the Lower Anogenital Squamous Terminology (LAST) Project recommended a uniform two-tiered terminology for human papillomavirus (HPV)-associated squamous intraepithelial lesions (SIL) across all anogenital tract organs.^2^ It distinguishes between low-grade SIL (LSIL) and high-grade SIL (HSIL). The World Health Organization (WHO) 2020 terminology for precancerous lesions of the vagina parallels that of other organs of the female genital tract. SIL is the preferred terminology, accompanied by a synonymous use of the three-tiered system of intraepithelial neoplasia. LSIL encompasses HPV infection and VaIN 1, while HSIL includes VaIN 2 and VaIN 3. A very small percentage of invasive squamous cell carcinomas of the cervix and vagina may develop independent of an HPV infection.^3,4^ The current WHO classification from 2020 has not included HPV-independent cervical and vaginal cancer precursor lesions due to lack of citable publications at the time of publication.^5^

### Colposcopic Terminology

Although the use of the colposcope is essential for the diagnosis of VaIN, the first colposcopic terminology of the vagina was published in 2011 by the International Federation for Cervical Pathology and Colposcopy (IFCPC)^6^ (Table 1).

**TABLE 1:** 2011 IFCPC Clinical/Colposcopic Terminology of the Vagina

General assessment	Adequate or inadequate for the reason (ie, inflammation, bleeding, scar) Transformation zone	
Normal colposcopic findings	Squamous epithelium: Mature Atrophic	
Abnormal colposcopic findings	General principles	Upper third/lower two-thirds, anterior/posterior/lateral (right or left)
	Grade 1 (minor)	Thin aceto-white epithelium, fine punctuation, fine mosaic
	Grade 2 (major)	Dense aceto-white epithelium, coarse punctuation, coarse mosaic
	Suspicious for invasion	Atypical vessels Additional signs: fragile vessels, irregular surface, exophytic lesion, necrosis, ulceration (necrotic), tumor/gross neoplasm
	Non-specific	Columnar epithelium (adenosis) lesion staining by Lugol’s solution (Schiller’s test): stained/non-stained, leukoplakia
Miscellaneous findings		Erosion (traumatic), condyloma, polyp, cyst, endometriosis, inflammation, vaginal stenosis, congenital transformation zone

Adapted from Bornstein et al.^6^

IFCPC, International Federation for Cervical Pathology and Colposcopy.

This nomenclature provides a standardized pattern recognition and interpretation. Furthermore, it distinguishes type 1 (minor) and type 2 (major) findings. Atypical and fragile vessels and lesions with an irregular surface and ulceration are suspicious for invasive disease. The reliability of the 2011 IFCPC vaginal colposcopic terminology is between 69.2% and 82.5%.^7–9^

To increase the reliability of the pre-biopsy colposcopic diagnosis, investigators proposed to add a micropapillary pattern category^8,9^ and negative Lugol’s iodine solution test (Schiller’s iodine test)^10,11^ to the abnormal colposcopic findings. A course of local estrogen therapy is given to post-menopausal women, as it may help to distinguish between benign mimics of atrophy and true pre-neoplastic changes.^12^

In contrast to the cervical colposcopic terminology, the consistency between colposcopic patterns of VaIN and histopathology has been reported to be less accurate, with the vaginal histopathology frequently being worse than what was anticipated by the colposcopic impression.^12,13^

### Epidemiology and Etiology of VaIN

VaIN (vaginal SIL) is a rare entity, accounting for only 0.4% of the female lower genital tract premalignant lesions. With an incidence of 0.2 to 2 per 100 000 women/year^14–17^ it is approximately 100 times less frequent than cervical intraepithelial neoplasia (CIN/SIL of the cervix).^18,19^ Despite a relatively stable incidence of vaginal cancers, the incidence rate of precursor lesions seems to have increased.^20,21^ This may be due to an improvement in the screening methods, as well as increased awareness of the condition.

The age-specific incidence rate of high-grade VaIN increases with advancing age until 70–79 years (1.5 per 100 000 woman years), after which it slightly declines. The incidence rate of high-grade vaginal lesions (VaIN 2/3; HSIL) was relatively stable during the total 20-year period but decreased significantly by nearly 16% per year among the youngest individuals (<30 years) in the period following licensure of the HPV vaccine.^22^

### HPV Infection and Oncogenesis

The large majority of vaginal neoplasms are HPV-associated squamous cell carcinomas that develop through VaIN (vaginal SIL). Low grade lesions of the vagina (VaIN 1; vaginal LSIL) are associated with both low-risk and high-risk HPV genotypes. In vaginal high-grade lesions (VaIN 2/3; vaginal HSIL), the most common genotypes involved are: HPV 16, HPV 33, and HPV 45.^23^ Individuals with risk factors for persistent HPV infection (eg, smoking, immunosuppression, HIV infection, history of cervical HSIL) have an increased risk for vaginal precancerous lesions and cancer,^24–27^ as well as vulvar, perianal, and anal lesions. The reported progression rate of VaIN towards invasive squamous cancer ranges between 2% and 7%.^27–29^

Vaginal precancer/cancer is also known to occur more frequently in patients with a history of pelvic radiation for other malignancies, such as cervical or endometrial cancer.^30^ The mechanism of HPV-independent carcinogenesis of the vagina is unknown.^5^ Vaginal adenosis may be the origin of the rare entities of vaginal adenoma and adenocarcinoma.^31^

### Genetics of VaIN

Little is known about the genetic risk factors for VaIN and vaginal cancer. Based on the current studies, no gene mutations associated with hereditary forms of vaginal cancer have been identified.

The persistence, progression, or regression of the HPV-induced lesions may depend, among other factors, on the host heritable immune response.^32^ Genetic factors may influence the susceptibility to cervical high-risk HPV (hr-HPV) infection.^33,34^ There are no specific studies of this kind concerning VaIN, but it is likely to be similar to what is known for the cervix.

Over the last three decades, numerous studies on gene association, using either the candidate gene approach or genome-wide association studies (GWAS), have been conducted in an attempt to identify genetic factors associated with persistent HPV infection and cancer development. It is suggested that a disruption in the apoptotic and immune function pathways plays a key role in the susceptibility to HPV-associated cancers.^35^ Epigenetic and in particular differential methylation events substantially contribute to the regulation of the papillomavirus life cycle.^36^ Methylated genes (CpG sites for cell adhesion molecule 1 (CADM1)), T-lymphocyte maturation associated protein (MAL), and the microRNA 124–2 (miR124-2) appeared to be promising biomarkers in HPV-related CIN.^37^ As indicative of underlying biological changes, they might become useful as markers of neoplastic transformation at other lower genital tract sites.^38^

### Vaginal Microbiome

Stability and composition of the vaginal microbiome plays an important role in determining host innate immune response and susceptibility to infections, including HPV. Depletion of *Lactobacillus* species has been associated with the presence of hr-HPV infection and increases with disease severity.^39–43^ The rate of a *Lactobacillus*-depleted microbiome is only 10% in healthy individuals, while this increases two-, three- and four-fold in patients with CIN 1 (LSIL of the cervix), CIN 2/3 (HSIL of the cervix) and invasive cervical cancer, respectively.^40,44,45^ Furthermore, *Lactobacillus* depletion has been found to be associated with CIN progression or regression.^46^ This high diversity microbiome persists after surgical excision of CIN and HPV clearance, suggesting that this microenvironment may contribute to the susceptibility to HPV and is not caused by the infection.^47^ Similarly, patients with VaIN have increased abundance of several bacterial vaginosis-related bacteria.^44^

Potential mechanisms of vaginal microbiome influence are through changes in vaginal pH, bacteriocin production, mucosal disruption and epithelial integrity, oxidative stress, and effects on cellular targets such as p53, pRB, and survivin, synergistically with HPV.^48^ Future research on vaginal microbiota may reveal new important information for understanding the onset and biological behavior of VaIN.

### Cytology, Histopathology, and Immunochemistry

While cytology can be helpful in the detection of vaginal pre-invasive lesions, in the individual who still has a cervix, the finding of dysplastic squamous cells, or metaplastic or glandular cells, does not necessarily indicate a diagnosis of VaIN or adenosis, as cervical contamination is possible. Thus, cytology is not considered a primary screening modality for these conditions. Vaginal cytology may also be utilized after therapy for follow-up of a treated vaginal lesion, as well as for follow-up of cervical pre-invasive and invasive disease in a patient who had a hysterectomy.

Immunohistochemistry is a useful tool for distinguishing between different types of vaginal pre-invasive lesions (Table 2).

**TABLE 2:** Immunohistochemistry in Vaginal Pre-invasive Lesions

Lesion	Histochemistry/immunohistochemistry	Comment
HSIL (VaIN 2/3)	p16 block positivity, Ki-67 extends above basal layers through the entire epithelium. p63 and p40 will confirm squamous origin, if in doubt	Ki-67 will stain above the basal layers in LSIL as well and cannot be used to distinguish LSIL from HSIL. p16 is more useful in this distinction.
Adenosis	Mucicarmine or periodic acid shift (PAS reaction) with and without diastase will highlight mucin producing cells	
Pagetoid spread of urothelial intraepithelial neoplasia	Positive cytokeratin 7, cytokeratin 20, p63, and GATA3 staining^149^ and uroplakin^150^	Exceptionally rare
Paget disease	Cells are positive for PAS-D, mucicarmine, CK 7, GCDFP-15, GATA3^151^	Exceptionally rare. Stains to distinguish secondary Paget disease of urothelial (including uroplakin^150^) or anorectal origin (including CDX-2, CK20^152^) should be considered in appropriate cases
Melanoma in situ	Positivity for s100, Melan-A, and HMB 45^153^	Exceptionally rare. A panel to distinguish melanoma *in situ* from Paget disease can be helpful

HSIL, high-grade squamous intraepithelial lesions; LSIL, low-grade squamous intraepithelial lesions; VaIN, vaginal intraepithelial neoplasia.

High-grade VaIN (VaIN 2/3, vaginal HSIL) is most often found in association with previous or current cervical neoplasia,^49^ and is cytologically and histologically identical to that of the vulva and cervix. Cytology focuses on increased nuclear to cytoplasmic ratio with irregular hyperchromatic nuclei. Histology shows maturation abnormality of the squamous epithelium at least two-thirds of the way up from the basement membrane. As in vulvar and cervical HSIL, the neoplastic cells have hyperchromatic irregular nuclei, and mitotic figures are often seen. p16 block positivity is an indicator of transforming hr-HPV infection and can be used to distinguish HSIL from its mimics, although LSIL is occasionally associated with hr-HPV and might show block positivity. Ki-67 immunohistochemistry will distinguish SIL from non-SIL, by extending above the basal layer, but this staining pattern will not distinguish LSIL from HSIL. Neither LAST^2^ nor the 2018 International Federation of Gynecology and Obstetrics (FIGO) staging for vaginal cancer describes criteria for a superficially invasive lesion.^50^

### Clinical Aspects

Vaginal intraepithelial neoplasia is an underdiagnosed disease. Due to the absence of symptoms, it is more often diagnosed after a positive cervical cytology and/or HPV test in the absence of cervical intraepithelial neoplasia on colposcopy and/or biopsy, or during follow-up of patients previously treated for cervical disease.

Individuals at higher risk for development of VaIN are those:

- With a history of cervical cancer or cervical HSIL^51,52^
- Who had a hysterectomy for cervical HSIL^53^
- Who had previous irradiation for gynecological cancer^30^
- Immunosuppressed individuals^54^
- Post-menopausal individuals^51^
- Diethylstilbestrol (DES) exposed patients.^55^

#### Cytology

Most vaginal lesions are diagnosed as a result of an abnormal cervical screening test. Individuals who have a positive cytology in the absence of cervical pathology should be surveyed for the presence of vaginal lesions. Cytology is sensitive (67.5–76.2%) and more reliable than colposcopy for detecting vaginal lesions.^56^ When combined with hr-HPV tests, it can improve detection accuracy up to 95%.^57^

#### Colposcopic Assessment of the Vagina

**FIGURE 1:**
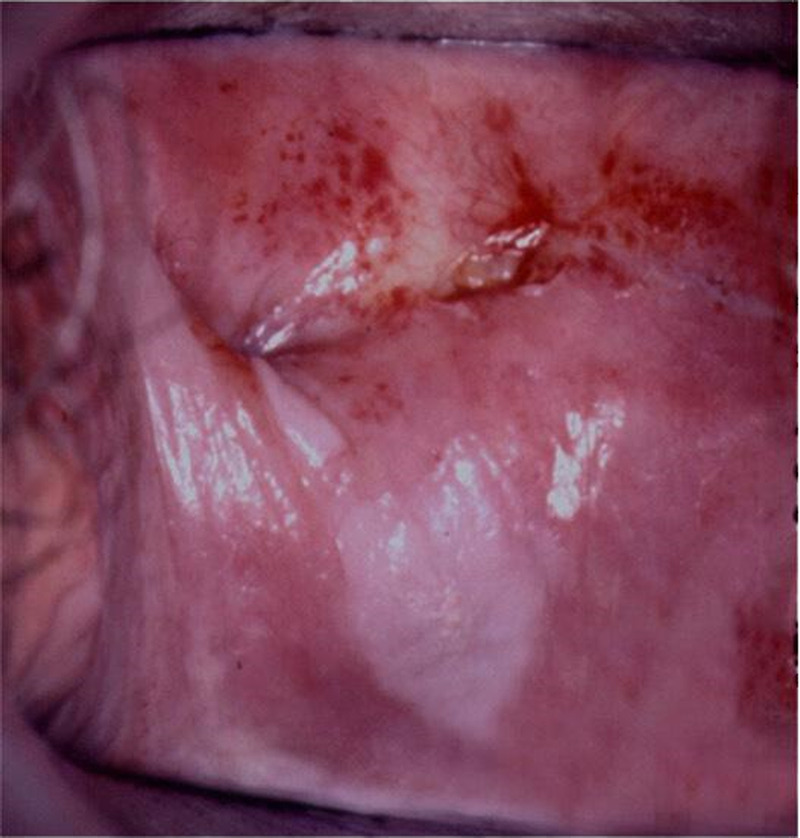
VaIN 3 (vaginal HSIL) on the posterior vaginal wall and between folds of the vaginal cuff. HSIL, high-grade squamous intraepithelial lesions; VaIN, vaginal intraepithelial neoplasia.

There is often no gross identifiable lesion in the vagina during visual inspection. Therefore, the examination of the vagina using a colposcope is essential. It requires not only the usual application of 5% acetic acid, it must include the complete visualization of the vaginal walls and folds. The vaginal folds make it difficult to detect all suspicious areas because the lesions may be hidden between the mucosal folds of the vagina and between the cervix and vaginal fornices (Figure 1). When undertaking examination, it is important to rotate the speculum with the blades opened through 360 grades.

Colposcopic assessment of the vagina is complicated by several problems:

- The field to be examined is large
- It is difficult to see most of the changes at a right angle
- The colposcopic patterns can be less specific than in the cervix
- Following hysterectomy, affected areas may not be readily visible at the oversewn vaginal vault including the lateral ‘dog-ears’
- Pre-invasive disease is often multifocal
- It is important to differentiate LSIL from truly premalignant lesions (HSIL) to avoid overtreatment.

**FIGURE 2:**
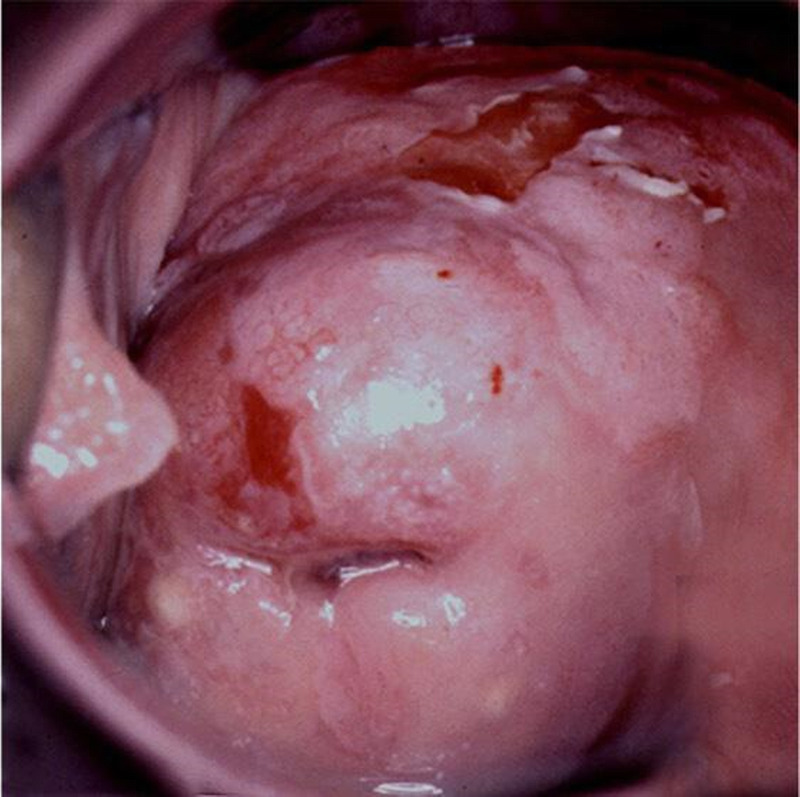
CIN3 (cervical HSIL) extending to anterior vaginal wall (VaIN 3/vaginal HSIL). CIN, cervical intraepithelial neoplasia; HSIL, high-grade squamous intraepithelial lesions; VaIN, vaginal intraepithelial neoplasia.

After the application of acetic acid, vaginal HSIL is usually aceto-white with sharp borders and a granular surface appearance. Occasionally, a punctation pattern can be seen. Mosaic or keratosis are rarely found (Figure 2).

**FIGURE 3:**
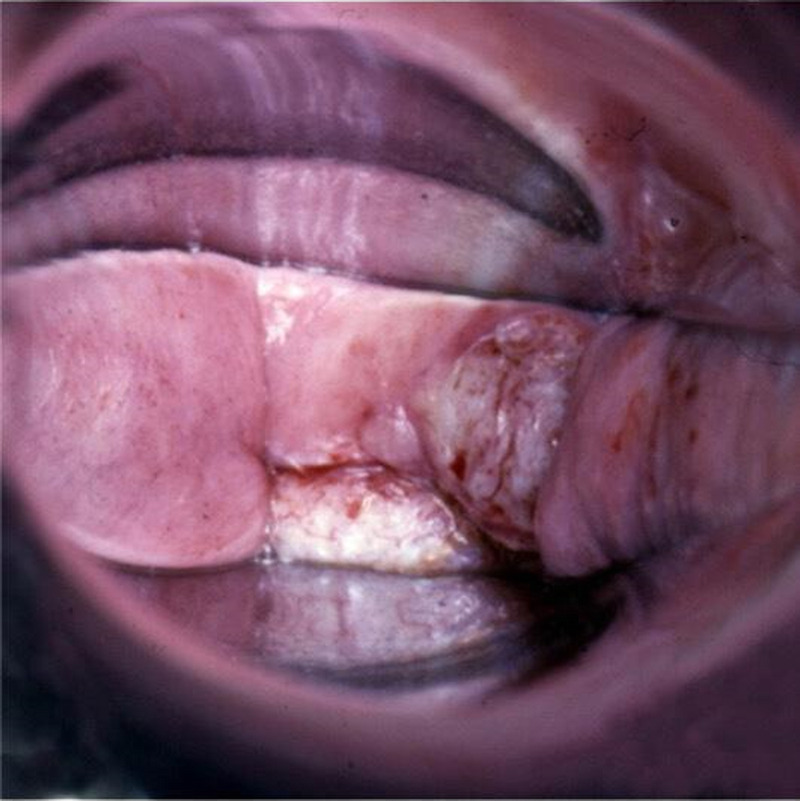
Invasive cancer at vaginal cuff after hysterectomy for cervical HSIL. HSIL, high-grade squamous intraepithelial lesions.

Similar to other sites, atypical and fragile vessels, and lesions with an irregular surface and ulceration, are suspicious for invasive disease^58^ (Figure 3).

**FIGURE 4:**
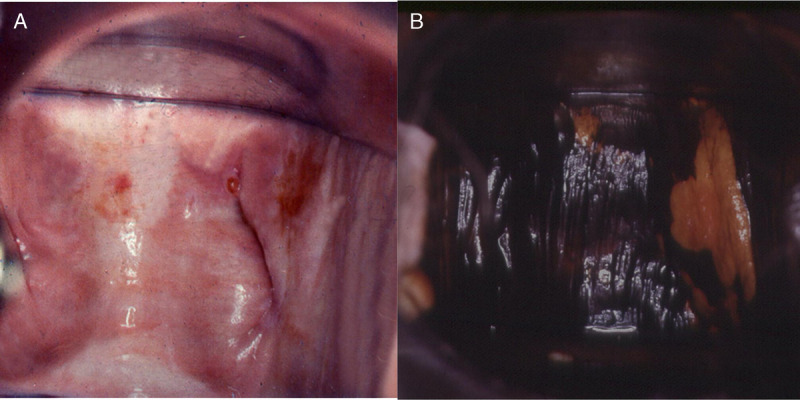
VaIN 3 (vaginal HSIL) (A) after the application of acetic acid, and (B) after staining with Lugol’s solution. HSIL, high-grade squamous intraepithelial lesions; VaIN, vaginal intraepithelial neoplasia.

The application of Lugol’s iodine solution (Schiller’s test) is important in colposcopy of the vagina. Colposcopically, VaIN may present as iodine-negative epithelium only, similar to what is observed on the cervix in some cases^58^ (Figure 4). In post-menopausal patients with a marked atrophy of the vaginal mucosa the interpretation of Schiller’s iodine test may be difficult. The application of a topical estrogen for up to 3–4 weeks before the exam is recommended.

Histology obtained by biopsy is the gold standard for the diagnosis. The 2020 WHO classification is used to determine the treatment.^5^

### Management

There is no unanimous agreement on which is the best method of the treatment of VaIN. Treatment should be individualized, based on characteristics of each patient, disease, and previous therapeutic procedures. The choice of the treatment depends on:

- Patient characteristics (age, parity, immune status, sexual activity)
- Type of the lesion (severity and site of the lesion, extent of the disease, multicentricity)
- Previous treatment (treatment of VaIN, hysterectomy for HSIL of the cervix, previous irradiation).

Vaginal atrophy may create diagnostic difficulties related to colposcopic assessment and may be the cause of overreading of vaginal cytology. Therefore, histological confirmation of vaginal neoplasia is necessary before treatment planning.

Low grade lesions of the vagina (HPV changes/VaIN 1; vaginal LSIL) are considered expressions of HPV infection with a low risk for progression and a high potential for spontaneous regression. Studies including the observational approach of VaIN 1 have shown that it spontaneously regressed without treatment in 48.8–88% of cases.^57,59–62^ Lesions not associated with HSIL of the cervix or vulva tend to have higher spontaneous regression (91%) than those associated with cervical or vulvar HSIL (67%), suggesting different biologic behavior.^61^

There is evidence that treatment does not lead to better clinical outcomes in patients with VaIN 1.^63^ As such, low grade lesions can be safely managed by observation.^64^ Continuous surveillance is warranted due to the frequent emergence of recurrence even after treatment with laser or excision (24.3% and 22.2%, respectively).^59^

High-grade lesions of the vagina (VaIN 2/3; vaginal HSIL) have premalignant potential and should be treated. Studies of patients with HSIL of the vagina who were monitored without any treatment reported progression to invasive cancer ranging from 9%^61^ to 50% of cases.^65^

A wide spectrum of modalities has been used to treat VaIN. Traditional methods, vaginectomy and vaginal irradiation, are nowadays used only in highly selected cases of extensive and persistent disease. Both treatments cause significant morbidity that greatly worsen the quality of life.^14^ More conservative options such as local excision, laser ablation, and medical therapy with topical agents are useful as first line treatments, especially in young patients and for multifocal disease. Conservative treatment aims to ensure maintenance of the functional anatomic structure, preserving the elasticity, capacity, and extension of the vagina. Each treatment modality has advantages and disadvantages to be discussed with the individual patient.

### Surgical Interventions

Surgical methods used for treatment of VaIN include both excisional and ablative techniques. Cold knife, carbon dioxide (CO_2_) laser, cavitational ultrasonic surgical aspiration, and electrosurgical loop excision are usually used for excision, while CO_2_ laser vaporization, photodynamic therapy, and electrocoagulation (fulguration) have an ablative effect.

#### Excisional Methods

Excisional methods are preferred because they provide a specimen for a complete histopathological diagnosis and permit the identification of underlying invasive cancer. Pre-operative colposcopic assessment of the vagina to identify the extent of VaIN should be done to ensure adequate excision and avoid residual disease. The uneven surface of the vagina makes it difficult to accurately assess the length of surrounding tissue to be removed.

Wide local excision is associated with the lowest risk of recurrence, but it is limited in applicability because SIL of the vagina is frequently multifocal. The reported residual disease rate after excision ranges from 8.6%^66^ to 18.9%.^67^

The success rate after surgical excision of VaIN is high, ranging between 66% and 81%. In a study of 35 patients with VaIN 3 treated by wide local excision, 23 patients (66%) were free from disease at a median follow-up of 44 months.^66^ More recently, a study exploring the outcome of ‘vaginal stripping’ in VaIN 3 found that 90 out of 111 (81%) patients were disease-free after a median follow-up of 76 months. The vaginal stripping procedure was performed as the combination of sharp and blunt dissection used for en bloc removal of the mucosa of the upper vagina, followed by cauterization to achieve hemostasis. Apart from short-term complications such as hemorrhage or infection evidenced in 4% of patients,^67^ this procedure may result in other complications which include shortening or stenosis of the vagina.

CO_2_ laser therapy is used for both local tissue excision and ablation. This method enables easier treatment of multifocal disease with limited morbidity. Pain and bleeding are the most frequent complications. In a large retrospective series of 128 cases of VaIN 3 treated with CO_2_ laser excision only, the overall rate of complication was 7.8% (mostly vaginal bleeding). There was only one (0.8%) major complication (vaginal vault perforation).^68^ Laser excision is usually combined with other modalities for treatment of VaIN. Laser excision should be performed only by expert specialists to avoid tissue damage and intra-/post-operative complications.^17^

Partial upper vaginectomy is considered the treatment of choice in high-grade VaIN (vaginal HSIL) at the apical part or in the region of the vaginal cuff scar.^69^ In cases of multifocal lesions or those that involve the lower one-third of the vagina, upper vaginectomy can be combined with laser vaporization.^70^

In a retrospective review of 33 patients with VaIN 2/VaIN 3 extending between 20–100% of the vaginal surface treated by single laser skinning vaginectomy, Luyten et al achieved a cure rate of 87.0%. The vaginal epithelium, including all lesions, was excised in one piece with a depth of 2–3 mm. No serious adverse events related to the procedure were recorded. After follow-up of 23 patients for at least 12 months, moderate shortening of the vagina was observed in two patients and another two required treatment of vaginal strictures.^71^

Similar cure rates were reported in two studies of patients who underwent partial (upper) vaginectomy for VaIN 3, 84% and 88%, respectively.^70,72^ Post-operative complications ranged from none to 3.5%.

Total vaginectomy is not an advisable procedure because it makes sexual intercourse impossible and thus it must be reserved for exceptional cases, when the spread of recurrent lesions cannot otherwise be managed or in cases of a short vagina post-hysterectomy. The complications of total vaginectomy could be decreased with adequate patient selection and meticulous surgical procedure.^73^

Cavitational ultrasonic surgical aspiration (CUSA) is a safe and effective option for VaIN, with effectiveness similar to classical surgery. Ultrasonic surgery allows exact removal of epidermal or mucosal lesions without thermal or mechanical damage to the surrounding structures or underlying stroma. It is a minimally-invasive procedure which requires general or spinal anesthesia. However, CUSA requires expensive equipment, training, and is not available in most settings.

After a median follow-up period of 4.5 years, the cure rate in 92 patients who underwent CUSA for VaIN was 80.4%.^74^ There are no reports of adverse effects in patients treated with CUSA.

In a study involving 46 patients treated with CUSA for recurrent disease, a significantly greater proportion of those who were treated with CUSA had no further recurrence (52%) compared with patients treated with other methods (9%) (p<0.001).^75^ Similar effectiveness was reported for the treatment of recurrent disease (50%).^74^

The loop electrosurgical excision procedure is not a treatment of choice for vaginal lesions due to the difficulty in controlling the depth of excision. Deep necrosis is reported as one of the possible late complications.^76^

Still, the loop electrosurgical excision procedure has been reported in treating upper vaginal vault VaIN.^77^ When the loop electrosurgical excision procedure was used for the treatment of 23 patients with histologically confirmed VaIN (VaIN 1–3) a complete response rate of 86.96% at 12 months of follow-up was reported, while at 24 months of follow-up it was 75%.^78^ The advantage over knife, laser, or diathermy excision is not clear.

#### Ablative Methods

The major disadvantage of using ablative methods for the treatment of VaIN is the risk of missing an invasive cancer, since they do not provide tissue specimens for histopathological evaluation. Occult invasive cancer has been reported in 2.6–30% of patients.^16,62,67,68,71,79^

**FIGURE 5:**
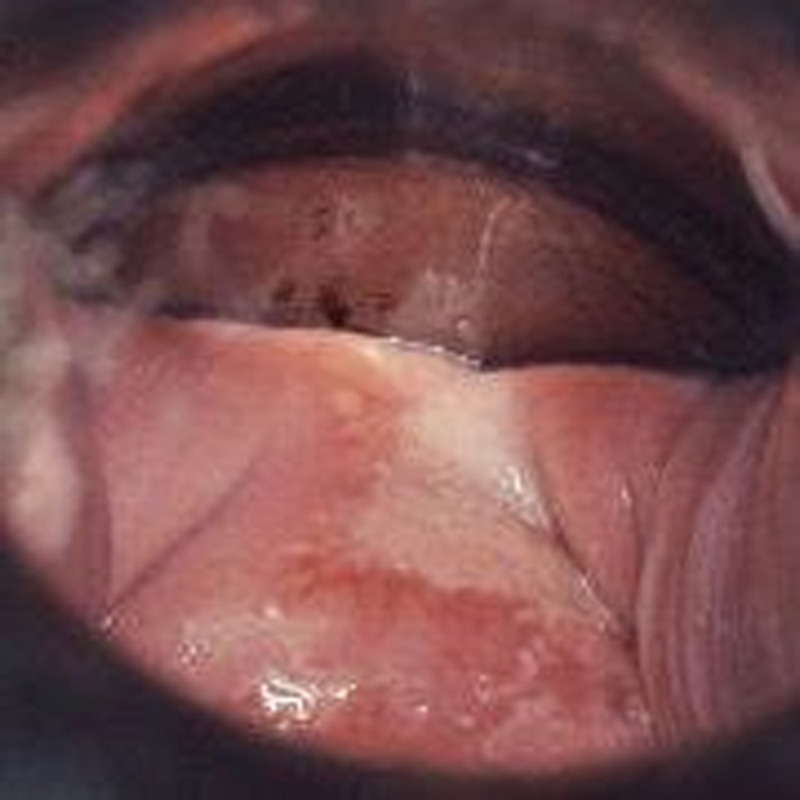
VaIN 3 (HSIL of the vagina) buried within the vaginal cuff. HSIL, high-grade squamous intraepithelial lesions; VaIN, vaginal intraepithelial neoplasia.

Special attention is needed in patients with prior hysterectomy for cervical HSIL extending to the upper vaginal vault scar. In these cases, a buried residual lesion (VaIN or occult cancer) cannot be reached by local ablative treatment (Figure 5).

For this reason, ablation should not be performed if the entire area of abnormal epithelium cannot be visualized or if there is any suspicion of invasion on colposcopic assessment, with multiple biopsies recommended before ablation to rule out an invasion.

When using ablative techniques an attempt should be made to achieve a depth of destruction to include epithelium affected by VaIN, because it directly relates with the outcome of the treatment. Exploring the depth of the involved and non-involved vaginal epithelium in 246 patients with VaIN, Cui et al found that the thickness of the lesion was generally <1 mm for patients of all ages, except in rare cases of visible lesions with papillary hyperplasia. The mean thickness of the epithelium involved was 0.4 mm and it did not differ between the grades of VaIN.^80^

CO_2_ laser vaporization can be performed under local anesthesia. Epithelial destruction to a depth of 1–1.5 mm, including the zone of thermal necrosis, seems to be sufficient to destroy the epithelium containing SIL, without damage to the underlying structures. Larger spot sizes and the superpulse mode are used to avoid deep penetration and the conduction of excessive heat.

Reporting the treatment of 65 patients with all grades of VaIN by laser vaporization, Jentsche et al confirmed relapse of the disease in 57 % cases.^81^ Cure rate in patients treated once with CO_2_ laser vaporization for high grade (VaIN 2/3) is between 73.5%^59^ and 86%.^82–84^ In a study involving 24 patients with VaIN 3, the lesion was completely eliminated in 17 (70.8%) patients after one session of treatment, and 19 (79.2%) required multiple sessions.^85^

Disadvantages of laser vaporization include an inability to treat buried vaginal cuff epithelium and technical difficulties in applying the laser to a distorted space within vaginal foldings. Also, expensive equipment, technical support, and surgical expertise are required, which are not easily available in all centers.

Photodynamic therapy (PDT) is an ablative, highly selective, and effective method for treating intraepithelial lesions and HPV lesions of the lower genital organs. It combines a medical and physical approach, which relies on a photosensitizer. The photosensitizer (5-aminolevulinic acid (ALA)) is selectively absorbed by abnormal cells and can be activated by light at a specific wavelength to produce singlet oxygen, which kills the target cells.^86,87^ Only mild local adverse reactions were recorded (burning sensation, pain, slight discomfort in the lower abdomen, and increased vaginal discharge), which were bearable and resolved in 3–5 days after treatment. At the end of follow-up, both the cervix and the vagina of all patients had maintained their integrity with regards to anatomical structure and function.^88^

Evaluation of the efficacy and safety of ALA-PDT for treatment of hr-HPV-positive patients diagnosed with VaIN showed complete remission rates ranging between 88.64% and 90.9%.^86,88,89^ The HPV clearance rate ranged from 38.1%^89^ to 60.98% and 67.1% at 12 months of follow-up.^86,88^

Electrocoagulation (fulguration) has also been used in the therapy of VaIN. Diathermy can reach and control the desired depth of ablation of 1.5 mm. However, it is less precise than laser.

A retrospective study of 184 patients with VaIN whose main treatment was electrofulguration with focal resection showed a primary remission rate of 87.62%.^90^ It was also safe, with few complications. Some patients complained of discomfort after surgery.

Plasma energy ablation is an ablative technique which vaporizes tissues, similarly to CO_2_ laser ablation, with advantages in terms of safety and the need for training and expertise. Kinetic and thermal energy generated by this technology can dissect, vaporize, and coagulate tissue, in the same manner as the CO_2_ laser. In contrast with laser, the energy transferred with plasma ablation decreases rapidly with increased distance of the handpiece to the tissue, significantly reducing the risk of both fire and retinal injury.

After a median follow-up of 29.3 months of 41 patients treated for vulvar or vaginal HSIL, a similar rate of complications (4.8% vs 9.5%) and recurrence rates (33.3% vs 28.6%) were reported in the plasma and laser ablation groups.^91^ Plasma energy ablation is considered a viable alternative to CO_2_ laser ablation, which may be particularly important in countries with limited access to the latter.

Finally, proper selection of patients, but also the skills of the surgeon, have a significant influence on the outcome.^62,92^

### Medical Therapy

Topical application of therapeutic agents has the advantage of treating the entire vaginal mucosa with good coverage of multifocal disease and disease in folds and recesses of the vagina. However, local vaginal creams cannot reach buried epithelium in the vaginal cuff scar. Also the effect on the lower vagina may not be consistent when the cream is applied using a standard vaginal applicator. As with ablative methods, prior to medical treatment invasion must be ruled out.

Imiquimod is an immune response modifier that induces cytokines which stimulate the activity of natural killer cells, promotes maturation and activity of Langerhans cells, and increases the effectiveness of T-cell-mediated response.^16,93^

Being proved useful in the treatment of vulvar intraepithelial neoplasia, imiquimod has recently gained much interest for the treatment of vaginal lesions. Applied for persistent HPV infection after treatment of cervical or vaginal SIL (VaIN), after a median follow-up of 33.6 months, imiquimod led to cytological/histological regression and negative HPV in 51.4% of the 72 treated patients.^94^ Of the 26 patients with normal cytology but persistently HPV-positive tests for at least 1 year, a complete regression was achieved in 65.4%. Chen et al reported an even higher clearance rate of HPV: 76.3% of the 76 patients cleared the HPV infection and had a normal cytology following the use of imiquimod cream.^93^ In most cases of persistent HPV infection, the severity/grade of VaIN decreased following the use of imiquimod.

A very low dosing regimen of imiquimod 5% cream (0.25 g, once a week for 3 weeks) appeared to be an effective and well-tolerated treatment for low grade VaIN. Thirty-six of 42 (86%) patients from a study by Buck et al achieved clearance of vaginal lesions on completion of the initial course of treatment. After the follow-up for at least 6 months, 92% of patients remained clear of VaIN.^95^

Results from a randomized clinical trial showed that vaginal imiquimod appeared to be as effective as laser treatment for the treatment of VaIN. Histological regression was observed in 80% of the cases in the imiquimod arm, 100% in the laser arm, and 67% in the expectant management arm (p=0.628).^96^

The most recent systematic review, including 28 patients from five articles and nine cases of VaIN 2/3 treated with imiquimod, reported a pooled complete response rate of 76% and a response rate of 89%, regardless of a history of hysterectomy.^97^ The authors concluded that imiquimod seemed effective for treatment of VaIN 2/3. The treatment itself is demanding since it must be carried out at least three times a week for 8 weeks and requires a significant commitment by health professionals. However, self-administered vaginal imiquimod, as used in a randomized prospective study by Tainio et al, appeared to be an acceptable mode of treatment, which would certainly lead to a better compliance from patients^96^

5-Fluorouracil (5-FU) was considered promising in the local therapy of VaIN. In a study by Fiascone et al, 104 patients with VaIN were treated initially with 5-FU, excision, or laser ablation. Patients who received 5-FU had the highest cure rate (74% compared with 57% and 41%, respectively).^98^ An even higher cure rate (81–86%) was shown in another study involving 30 patients treated with 5-FU.^99^ On the other hand, there are studies reporting lower cure rates, such as 62.5%.^100^ Among patients treated with 5-FU for recurrence of VaIN, 62% did not experience a second recurrence.^98^ Although treatment with 5-FU is effective, its local side-effects including vaginal discharge, burning, pain or ulcers may be highly uncomfortable and reduce compliance. Approximately 16% of patients treated with 5-FU reported a side effect, most commonly irritation and dyspareunia.^98^

Trichloroacetic acid, a powerful keratolytic agent confirmed to have a therapeutic effect on HPV-induced genital warts,^101^ was used in the past in an attempt to treat VaIN. Its use was abandoned as other effective types of treatment have emerged.

Rhodes at al evaluated the effectiveness of intravaginal estrogen therapy as a potential primary treatment modality for VaIN. In a study involving 83 patients with VaIN 1–3 treated by different modalities with or without additional local estrogen, the overall regression rate was 85.5%. In the group of 40 patients treated with intravaginal estrogen only, 90% had regression or cure.^102^ At the same time, 32 patients who underwent treatment with intravaginal estrogen in addition to one or more other treatment modalities experienced regression or cure in 81.3% of cases, while in patients undergoing treatment without intravaginal estrogen, lesions regressed in only 71.4% of cases.^102^

### Radiotherapy

External beam radiotherapy is not indicated for the treatment of VaIN. Brachytherapy is a good option, though it is usually not proposed as the first-line therapy because of the risks of long-term radiation effects. It also compromises the possibility for secondary surgery in case of recurrence and makes the colposcopic examination extremely difficult. Nevertheless, it may be effective for selected patients with VaIN 2/3 whose disease relapsed after conservative therapies or with conservative surgery not being feasible. Both low-dose rate and high-dose rate intracavitary brachytherapy have been used for treatment of VaIN. There is no standardization of dose prescription in VaIN and patients should be referred to expert centers. The common dose prescription is 60 Gy to 5 mm below the surface of the vaginal mucosa, delivered through continuous low dose rate brachytherapy or pulse dose rate brachytherapy. Higher doses may cause significant vaginal fibrosis and stenosis. No studies comparing low-dose rate with high-dose rate have been carried out with respect to outcomes and acute and late toxicities in VaIN. Apparently, no differences exist between these two techniques when used for treatment of vaginal invasive cancers, provided that the total dose is reduced to take into account hypofractionation.^103^

In a retrospective study, 28 patients with VaIN were treated by low-dose rate brachytherapy, using a personalized vaginal mold delivering 60 Gy to 5 mm below the vaginal mucosa. After a median follow-up of 41 months, only one ‘in field’ recurrence occurred, corresponding to a 5- and 10-year local control rate of 93%.^104^ A disease-free survival rate in other studies is similarly high at 86.37–90%.^105^

Brachytherapy for VaIN is usually well tolerated. Reported acute toxicity was minimal.^106^ After a median follow-up of 48 months, 44% of 34 of patients from the series by Song et al had experienced toxicity, predominantly vaginal mucosal reaction. In this series, 27/34 patients had received 40 Gy through eight fractions of 5 Gy high-dose rate, with radiation prescription points ranging from 0 to 5 mm from the surface.^106^ Late consequences of brachytherapy include alterations in vaginal depth and diameter, elasticity, sexual function, and overall quality of life. Zolciah-Swinska et al reported a series of 20 patients who were treated with brachytherapy, in which the most frequent late complications were dyspareunia (35%) and stenosis grades 2–3 (35%).^105^ In another study, after a median follow-up of 77 months, five out of 20 patients experienced G3 toxicity, predominantly stenosis of the vagina, and one case of G4 toxicity resulting in vaginal ulceration.^107^ No second cancers were reported after irradiation for VaIN.^104^

The potential sequelae of brachytherapy are to be weighed against the morbidity of total vaginectomy, especially in patients with extensive multifocal HSIL of the vagina (VaIN 2/3). Before brachytherapy treatment, it is mandatory to exclude invasive carcinoma through repeated biopsies and pelvic magnetic resonance imaging, as a primary invasive vaginal cancer would warrant discussing (chemo) radiation plus brachytherapy.

### Combination Therapies

In some studies, combination therapies for treatment of VaIN were used. Differences in combinations of treatment modalities, including all grades of VaIN in the analyses and low numbers of patients, make comparison of these studies difficult.

In the past, therapy with 5-FU was combined with microsurgery (so-called chemosurgery), particularly laser vaporization, expecting that the frequency of recurrences would be reduced. This approach nowadays has been abandoned due to its side-effects.^16^

For patients with recurrence of VaIN after surgical treatments, topical imiquimod with careful follow-up seems to be an effective and well-tolerated modality, with no apparent adverse events.^108^

Combining electrofulguration and focal resection in the treatment of 184 patients with VaIN, a primary remission rate of 87.62% was achieved.^90^ The same effectiveness was shown when combining excisional and medical treatments.^28^

Topical ALA-PDT combined with CO_2_ laser appeared to be an effective, safe, and well-tolerated treatment for vaginal LSIL and hr-HPV infections. In a study by Yao et al, which included 40 patients with vaginal LSIL and persistent hr-HPV infection, the complete remission rates were 65% in the CO_2_ laser group and 85% in the CO_2_ laser+PDT group (p>0.05). Remission rates of hr-HPV were 25% in the CO_2_ laser group and 95% in the CO_2_ laser+PDT group (p<0.05) at 1 year after treatment.^109^

### Risk for Recurrence after Treatment of VaIN

There is no consensus about the ideal treatment modality for VaIN. Different methods used for the treatment of VaIN, the small number of cases in some studies, combination of all grades of VaIN, and different duration of follow-up contribute to the wide range of reported recurrence rates. There are only a few randomized clinical studies that determined with a greater reliability which of the methods is the most successful for the treatment of VaIN. Therefore, most conclusions are drawn from individual cases series in which different treatment modalities were used.

Patients treated for VaIN are at high risk of developing recurrences. This depends not only on the method used, but on several other factors such as the grade of VaIN, localization, previous treatment, age, and immune status and consequently persistence of HPV infection.

#### Methods Used for Treatment of VaIN

The choice of the primary treatment might have an impact on further outcomes.^110^ A study on a large series of 132 patients with HSIL of the vagina treated by various modalities showed that the overall cure rate for excisional treatments and CO_2_ laser ablation was the same (69%).^62^ Less effective were 5-FU cream, which was curative in 46%, and electrocoagulation diathermy in only 25% of cases.

Comparison of different methods for treatment of vaginal HSIL showed different recurrence/progression rates (topical management 62.5%, laser ablation 26.4%, excision 32.7%, and radiotherapy 0%).^59^ The rates for surgical therapies, both excisional and ablative, were similar with 31% and 33%, respectively.^65^

In general, all excisional methods have similar recurrence rates which range from 7.2–20.8%.^67,72,74,83,85,110^ When comparing laser ablation and excision, similar local recurrence rates—17.1% in the excision group and 18.6% in the ablation group—were found, leading to the conclusion that the latter seems to be equivalent to excision in terms of long-term effectiveness.^110^ Other studies reported higher recurrence rates after laser vaporization (26.5–34%)^59,75^ and after CUSA (19.6–25%).^74,78^ Overall, recurrence rates comparing laser with CUSA were similar (25.5% and 24.4%, respectively).^111^

Recurrence rates are higher (61%) after medical treatment, compared with those after an excisional procedure (25%).^28^ Similar results were reported by Sopracordevole et al.^79^

Recurrence rates after irradiation are low, between 7.28%^106^ and 13.63%.^107^

#### Severity of VaIN

Although Zeligs et al reported that normalization, persistence, and recurrence rates did not differ by grade of dysplasia or treatment status,^60^ Lin et al found that severity of VaIN was the only significant independent predictor of persistence/recurrence (OR 3.5, 95% CI 1.1 to 11.6, p=0.038).^94^

In a large group of 576 patients with any grade of vaginal SIL, Kim et al noted spontaneous regression after observation in 48.8% of the patients with vaginal LSIL, compared with 46.2% in the vaginal HSIL patients.^59^ Patients with VaIN 2 who underwent treatment experienced recurrence or progression in 36.8% of cases,^59^ not much different from patients treated for VaIN 3 (38.5%).^57^ In another study among 131 patients, a relapse occurred in 15.26% of the patients with VaIN 3 and in only 3.05% of those with VaIN 2.^112^

#### Localization of the Lesion

Multifocal disease poses a treatment challenge. It has been shown that multifocal disease relapses more frequently (57%) than unifocal disease (43%).^92^

As the main risk factor for recurrence, HSIL (VaIN 2/3) in the vaginal vault was identified.^85^ Among 52 patients managed with laser ablation (28 patients) and upper vaginectomy (24 patients), cure rates of 68% and 80%, respectively, were achieved. The rate of failure of laser treatment in the hysterectomized group was twice that seen after upper vaginectomy (46% vs 20%).^92^

#### Previous Treatment

In a retrospective study of 118 patients with VaIN, Yu et al concluded that VaIN grade 2/3 and VaIN associated with CIN or cervical cancer are more likely to recur and progress to invasive cancer.^113^ Analysis of the medical history of 39 patients treated for VaIN with laser vaporization showed that patients diagnosed with VaIN after hysterectomy for high-grade CIN had a significantly higher success rate after the first episode of the treatment than patients who were previously treated for invasive cervical cancer (46.2% vs 0.0%).^82^ In a multivariate analysis of 375 patients who underwent hysterectomy and had a diagnosis of VaIN, it was shown that being aged over 50 was the only independent risk factor for recurrence of VaIN.^59^

#### Persistence of HPV Infection

One of the variables independently associated with a second recurrence is the persistent infection of HPV 16 or 18 (HR 3.87, 95% CI 1.15 to 13.0, p=0.028).^110^ High risk-HPV-positive VaIN was significantly more likely to relapse than hr-HPV-negative VaIN (p=0.005). There was also no significant influence in the relapse rate by VaIN grading, simultaneous CIN or vulvar intraepithelial neoplasia, previous therapy, or history of hysterectomy.^81^ In a retrospective review of 389 patients, those who underwent primary laser therapy, brachytherapy, or vaginectomy experienced high rates of remission in histopathologic follow-up (73.7%, 71.4%, and 100%, respectively) and similar rates of hr-HPV clearance (52.6%, 57.1%, and 50.0%, respectively).^114^

Laser vaporization appears not to be effective in eliminating HPV infection. After laser vaporization, HPV infection persisted in 61.8–89% of patients.^82,96^ In a study by Tanio et al, HPV clearance was significantly higher in the imiquimod arm (63%) than in the laser arm (11%) or in the expectant management arm (17%).^96^ HPV clearance rates after ALA-PDT treatment range from 38.1%^89^ to 60.98% and 67.1% at 12 months follow-up.^86,88^

### Progression to Invasive Cancer

The risk of VaIN progression to invasive vaginal cancer is not negligible. Invasive cancer after treatment of vaginal HSIL (VaIN 2/3) was reported to be 3.2% to 5.8% with a mean time interval from treatment to progression of 54.6 to 61 months.^59,79^ Jentsche et al reported that 6% of 65 patients with VaIN had developed vaginal cancer. All were hr-HPV-positive and all primarily had VaIN 3.^81^

In patients with VaIN 3 the rate of progression to invasive disease was significantly higher when compared with patients with VaIN 2 (15.4% vs 1.4%, p<0.0001). In other studies, even higher rates of progression of vaginal HSIL to invasive cancer were noted, ranging from 17% to 20%.^66,115^

### Follow-up and Surveillance Protocols

Similar to other HPV-related diseases, management of VaIN requires long-term follow-up, irrespective of the treatment modality, due to the risk of recurrence and progression to invasive squamous cell carcinoma, especially among those with VaIN 2 and VaIN 3 and prior hysterectomy for HPV-related disease.^17,63^

There is no consensus regarding the most adequate follow-up, following the treatment of vaginal SIL. There are varied follow-up practices in different centers. In general, it has been suggested to carry out follow-up schedules similar to those used for CIN. A negative HPV test and cytology (co-testing) can be considered a test of cure.

For follow-up of vaginal LSIL (VaIN 1) co-testing at 12 months is recommended. Given the high negative predictive value of HPV testing, only one co-test is needed. If only cytology is used it should be repeated at 12 months, two times. In case of a negative co-test or repeated cytology, further screening may be stopped. Patients with positive tests should be referred for colposcopy. For patients with persistent LSIL/VaIN 1 beyond 2 years without previous HSIL or cancer, it would be reasonable to extend the screening interval to every 2 to 3 years.

The meta-analysis of prevalence and type distribution of HPV in carcinoma and intraepithelial neoplasia of the vulva, vagina, and anus showed that HPV 6 and 11 were common in LSIL of the vulva and anus, but not in the vagina.^116^ In VaIN 1, HPV 16 predominated (23.4%), but a broad range of other HPV genotypes was detected, notably HPV 56 (11.0%) and 51 (8.8%). These results make the HPV test a useful addition to the follow-up of VaIN 1.

The first test after treatment of high-grade lesions of the vagina (VaIN 2/3) should be performed at 6 months with cytology and an HPV test, in order to avoid confusion with reparative phenomena. If there is a complete response to therapy and no new lesions at 6 months and 12 months follow-up, patients should be monitored by annual cytology or every 2–3 years co-testing.^117^ In the case of a positive HPV test and/or abnormal cytology, colposcopic assessment of the vagina is recommended. Colposcopy should be done by an experienced colposcopist. Abnormal colposcopic findings require biopsy.

VaIN may recur after several years, and therefore long-term follow-up is recommended. Because lower genital tract intraepithelial neoplasia is often multi-zonal, vulvoscopy and anoscopy in the presence of high-grade vulvar lesions should be considered during follow-up of patients treated for VaIN 2/3.^118^

### Prevention

Prevention of VaIN follows the principles for prevention of squamous intraepithelial neoplasia at other anogenital sites and presumes avoiding risk factors such as smoking, long-term oral contraception, multiple sexual partners, and unsafe sex. Persistent HPV infection, particularly by HPV 16, has been associated with the long-term development of HSIL (VaIN 2/3) and carcinoma of the vagina.^119^ Cigarette smoking cessation should be encouraged since, in combination with hr-HPV, it increases the risk of the development of vaginal HSIL when compared with non-smokers.^24^

Individuals with impaired immunity, including those with HIV infection, history of transplantation, and receiving immunosuppressive therapy,^120–123^ as well as patients previously treated for cervical HSIL, are under increased risk for vaginal precancer and cancer and should be under regular surveillance.

Education about regular gynecological examinations, particularly for high risk individuals, can help timely detection and treatment of vaginal precancer. However, prevention by education about avoiding risk factors and adopting a healthy lifestyle cannot be completely effective in eradicating the disease. The implementation of vaccination against HPV infection is expected to contribute to the prevention of VaIN, and thus, cancer of the vagina.

In clinical trials HPV vaccines were highly effective at preventing VaIN caused by vaccine genotypes.^124,125^ A recent cohort study from Denmark noted lower rates of vaginal HSIL among vaccinated compared with unvaccinated 17- to 26-year-old females (adjusted HR 0.3, 95% CI 0.13 to 0.68); the cumulative incidence of disease was low (as expected given the age of the cohort).^126^

The major risk factor for developing vaginal HSIL and invasive vaginal cancer is a history of HSIL of the cervix, especially when HPV 16 was the causal type.^127^ This risk persists after treatment. By now, there is no strong evidence that the risk for recurrent VaIN may be reduced by adjuvant HPV vaccination. HPV vaccination has been shown to reduce the risk for recurrence after treatment of cervical and anal intraepithelial neoplasia.^128^ While data for the protection of recurrent cervical HSIL are robust, numbers for vaginal disease are too small to draw final conclusions.

### Immunocompromised Patients

Immunocompromised patients encompass HIV-infected individuals, patients treated with immunosuppressive drugs, and those suffering from autoimmune diseases. These patients are at increased risk for acquisition and persistence of HPV infection,^129^ development of anogenital intraepithelial neoplasia, and progression to invasive HPV-related cancers.^54,130–135^ Given the low incidence of VaIN, the low prevalence of immunocompromised population, and the scientific interest for HPV persistence in the HIV-positive population, the only available evidence is about VaIN in HIV-infected patients.

HIV-positive patients show an incidence of VaIN of 0.2 per 100 person-years versus 0.01 per 100 person-years in HIV-negative individuals.^54^ In individuals living with HIV, VaIN presents at a median age of 39 years (vs 57 in HIV-negative), and is more frequently multifocal and multicentric.^136^ The strong HPV field effect in the whole lower genital tract is confirmed.^25,129,137^

Rates of recurrence and progression of VaIN in HIV-positive and HIV-negative individuals are reported to be 44.8% and 3.4%, respectively, over a median of 68 months follow-up. No risk factors were identified for recurrence or progression, despite a trend in those who were HIV-positive.^136^

Long-term follow-up of 335 post-hysterectomy patients (both for benign and malignant indications) found a 5-year clearance of abnormal cytology of 116/100 person-years in HIV-negative individuals and 34/100 person-years in HIV-positive patients,^54^ related to the severity of immunosuppression (CD4+ cells count^138^). However, most abnormal cytology reports reflect low grade disease or HPV transient infection, with 6.4% risk of ever having an HSIL or worse cytology over 12 years of observation.^54^ These data were confirmed in HIV-positive patients who underwent hysterectomy for benign conditions: among these patients, colposcopic-guided biopsy for abnormal cytology found VaIN in 29% cases,^139^ whereas in immunocompetent patients who underwent hysterectomy for benign conditions VaIN was found in only 0.1%.^140,141^

Annual vault cytology is recommended, by the Guidelines for the Prevention and Treatment of Opportunistic Infections in Adults and Adolescents with HIV, only for HIV-positive patients with a history of cervical HSIL, adenocarcinoma *in situ*, or invasive cancer.^142^ For individuals older than 65 years, it is recommended to continue screening because of the higher risk of HPV-related diseases.^142^ However, HIV-infected patients who underwent hysterectomy, even without a history of cervical lesion, should not be considered a low-risk population, and thus prolonged surveillance is mandatory to achieve early diagnosis of VaIN and proper treatment.

Low-grade SIL of the vagina (VaIN 1) should be observed. Treatment is considered for bulky warty disease depending on symptoms. For high-grade SIL of the vagina (VaIN 2/3) treatment should be tailored according to location, extension, and focality of the disease.^142,143^

There is no evidence concerning the best treatment of VaIN in immunocompromised patients. Topical treatments such as imiquimod or 5-FU are acceptable and have proved to be effective in an immunocompetent population,^97,144^ particularly in treating multicentric HPV-related diseases without aggressive and mutilating surgery. Cold-knife partial vaginectomy or CO_2_ laser skinning vaginectomy must be reserved for recurrent cases refractory to conservative management, as they are associated with important morbidities. Highly active antiretroviral therapy could have a positive impact also on the incidence and prognosis of VaIN, as already demonstrated for cervical, vulvar, and anal SIL,^134,135,145,146^ but further studies are needed to assess its impact.

### Education and Information

Patients with VaIN need clear and up-to-date information on the range of treatment options, including its risks and benefits, as well as the need for follow-up and risk of recurrence. Such information should improve decision-making and encourage attendance for surveillance post-treatment. However, no published trials or studies on this topic were identified in the performed literature search.

The internet is widely used as a source of health information which can help in increasing the awareness of current evidence and help decision-making. Online patient forums are an accessible source of help and mutual support. They allow patients to share their lived experience with others anywhere in the world. These forums allow an anonymous and non-judgemental environment which is important with vaginal disease, which is uncommon and a possible source of embarrassment.

Adherence to follow-up is particularly important for vaginal disease as VaIN has no signs or symptoms to alert the patient, and self-examination of the vagina is not possible.

### Qualıty of Life and Psychologıcal Sequelae of Vaginal Intraepithelial Neoplasia and its Treatment

While VaIN is symptomless and often only recognized in investigation or follow-up of cervical disease, the effects of treatment can have an impact on quality of life and result in psychological and psychosexual issues ranging from concerns around HPV infection and the risk of developing cancer to the after-effects of treatment.

Clinical surveillance on patients undergoing treatment for CIN and follow-up showed that while physicians consider risk as a reason for prevention, patients think of risk as ‘being sick’.^147^ Similarly, after treatment of VaIN, patients are faced with the potential risk of recurrence and the need for regular surveillance. They must deal with great uncertainty and perceive the process of ongoing gynecological review as an illness, which can affect their personal well-being and social relations.

Excisional treatments are associated with higher risks of sexual dysfunction, persistent pain, and scarring. Side effects of topical therapies include local burning and soreness which may interfere with usual activities. Radiotherapy, although uncommonly used for treating VaIN, may result in vaginal narrowing and atrophy. Studies of post-menopausal individuals clearly show that vaginal atrophy and dyspareunia are associated with a significantly higher incidence of depression, major depressive disorder, and anxiety.^148^ This observation may be well applied to patients after treatment of vaginal SIL.

Issues which have an impact on quality of life, including sexual function, need to be discussed with the patients when agreeing on treatment.^14^ Information and support from a specialist nurse should also involve the patient’s partner.

### CONSENSUS STATEMENTS

1. The management of VaIN varies according to the grade of the lesion: VaIN 1 (low grade vaginal SIL) can be subjected to follow-up, while VaIN 2/3 (high-grade vaginal SIL) should be treated. (Agreement 90%)
2. Treatment needs individualization according to the patient’s characteristics, disease extension, and previous therapeutic procedures. (Agreement 100%)
3. Surgical excision is the mainstay of treatment and should be performed if invasion cannot be excluded. Total vaginectomy is used only in highly selected cases of extensive and persistent disease. (Agreement 100%)
4. CO_2_ laser may be used as both an ablation method and excisional one. Reported cure rates after laser excision and laser ablation are similar. (Agreement 90%)
5. Topical agents are useful for persistent, multifocal lesions or for patients who cannot undergo surgical treatment. (Agreement 95%)
6. Imiquimod was associated with the lowest recurrence rate, highest HPV clearance, and can be considered the best topical medicament approach. (Agreement 100%)
7. Trichloroacetic acid and 5-fluorouracil are historical options and should be discouraged (Agreement 100%)
8. For VaIN after hysterectomy for CIN 3, laser vaporization and topical agents are not the best options, since they cannot reach epithelium buried in the vaginal scar. In these cases surgical options are preferable. (Agreement 100%)
9. Brachytherapy has a high overall success rate, but due to late side effects should be reserved for poor surgical candidates, those who have multifocal disease, and those who have failed prior treatments. (Agreement 100%)
10. VaIN tends to recur and ensuring patient adherence to close follow-up visits is of utmost importance. The first evaluation should be performed at 6 months with cytology and HPV test during 2 years and annually thereafter. (Agreement 100%)
11. The implementation of vaccination against HPV infection is expected to contribute to the prevention of VaIN, and thus cancer of the vagina. (Agreement 100%)
12. The effects of treatment can have an impact on quality of life and result in psychological and psychosexual issues which should be addressed. (Agreement 100%)
13. Patients with VaIN need clear and up-to-date information on a range of treatment options including risks and benefits, as well as the need for follow-up and the risk of recurrence. (Agreement 100%)
